# Not all is lost: resilience of microbiome samples to freezer failures and long-term storage

**DOI:** 10.1128/msphere.00603-24

**Published:** 2024-12-20

**Authors:** M. Fabiola Pulido Barriga, James W. J. Randolph, Sydney I. Glassman

**Affiliations:** 1 Department of Microbiology and Plant Pathology, University of California-Riverside, Riverside, California, USA; 2 Department of Ecology and Evolutionary Biology, University of California, Irvine, California, USA; University of Wisconsin-Madison, Madison, Wisconsin, USA

**Keywords:** freezer melt down, soil thawing, frozen DNA, DNA storage, richness, composition, bacteria, fungi

## Abstract

Advances in technology have facilitated extensive sample collection to explore microbiomes across diverse systems, leading to a growing reliance on ultracold freezers for storing both samples and extracted DNA. However, freezer malfunctions can jeopardize data integrity. To evaluate the impact of an unexpected −80°C freezer failure and the recoverability of thawed soil samples, we extracted DNA and compared it to long-term DNA stored at −20°C and original 16S and ITS2 sequencing data collected before the malfunction. Using Illumina MiSeq, we assessed how the freezer failure and long-term storage influenced the resilience of bacterial or fungal richness or community composition and our ability to accurately determine experimental treatment effects. Our results reveal substantial resilience in fungal richness and both bacterial and fungal beta-diversity to soil sample thawing and extended frozen DNA storage. This resilience facilitated biological inferences that closely mirrored those observed in the original study. Notably, fungi exhibited greater resilience to short-term thawing compared to bacteria, which showed sensitivity to both thawing and long-term freezing. Moreover, taxonomic composition analysis revealed the persistence of dominant microbial taxa under thawing and prolonged freezing, suggesting that dominant microbes remain viable for tracking across temporal studies. In conclusion, our study highlights that beta-diversity is more robust than alpha-diversity and fungi are more resilient to freezer failure than bacteria. Furthermore, our findings underscore the effectiveness of soil storage at −80°C compared to storage of extracted DNA at −20°C, despite potential freezer failures, as the most robust method for long-term storage in microbiome studies.

**IMPORTANCE:**

Microbiome studies heavily rely on ultracold freezers for sample storage. Unfortunately, these freezers are prone to frequent malfunctions, resulting in the loss of invaluable samples at laboratories worldwide. Such losses can halt research progress due to potential issues with sample reliability. Our research demonstrates that not all is lost when an unforeseen freezer failure occurs. Samples can still be reliably used to assess treatment effects, which is particularly important for long-term temporal studies where samples cannot be readily obtained again.

## INTRODUCTION

Advances in technology and affordability of molecular and “'omics”’-based techniques have led to a surge in microbiome research across systems including soils (1, 2), water (3), air (4, 5), atmosphere (6), smoke (7), animals (3, 8), and humans (9, 10). Yet, sample storage techniques can affect the diversity and taxonomic composition obtained from bacterial and fungal environmental (3, 11) and human samples (12, 13). Thus, microbiome research has adapted the methodology of extracting and storing microbial DNA at −20°C immediately after extraction (11) or storing environmental samples at −80°C when the latter is not feasible (3), to retain the microbial composition of fresh samples (14, 15). However, the increased dependency on freezers creates an added problem with sample storage: the potential loss of stored samples due to an unexpected freezer failure, which is particularly important for long-term studies investigating microbial community compositional changes over time. Understanding how freezer storage and failure can affect soil microbial samples is necessary to ensure that ecological conclusions are appropriately inferred when working with long-term studies or with previously frozen samples or DNA.

Soil sample storage temperature (16–18) and duration (16) can affect soil microbial diversity (16), activity (19), and taxonomic composition (20). While the current body of research lacks consensus regarding which microbial groups exhibit greater resilience, and under which conditions bacteria may be more resilient than fungi and vice versa, differences in microbial physiology suggest that bacterial samples are more resilient to small changes in temperature than fungi and might be less affected by variation in soil storage techniques or temperature (20, 21). Nevertheless, while fungi and bacteria can enter a dormant state under environmental stress (22), rapid growth can occur when the environment becomes favorable, such as with increased soil moisture resulting from soil thawing (22). Thus, the response of the soil microbes to thawing can potentially obscure signals of prospective studies. However, it is worth noting that at least one study suggests that variation between treatments was greater than variation among treatments for soil, human fecal, and human skin samples (15), implying that an unexpected freezer failure might not significantly affect the reliability of stored microbial samples when inferring experimental treatment effects.

The dependency on lab freezers for long-term storage of soil samples and extracted DNA for downstream analysis makes samples vulnerable to freezer failure. Given the propensity of freezer malfunctions in laboratories worldwide, it is critical to understand how short thawing events can impact microbiome samples, resulting in data loss or erroneous conclusions. Similarly, we must understand if long-term storage of frozen DNA can impact future ‘omics work. Here, we analyzed 20 soil samples that were previously sequenced using Illumina MiSeq technology targeting 16S and ITS2 regions to investigate bacterial and fungal diversity and composition (23). The DNA extracted from these previously sequenced samples had been stored for 2 years in an uncompromised −20°C freezer and excess soil from these samples was stored long term in a −80°C ultracold freezer that experienced a complete thaw event (~1 week) due to a freezer malfunction. This allowed us to compare the effects of short-term thawing of soil stored at −80°C vs the long-term storage of uncompromised extracted DNA stored at −20°C on the alpha and beta diversity of bacteria and fungi. It also facilitated our assessment of how these storage conditions influenced our ability to draw ecological conclusions based on treatment effects and taxonomic composition.

## MATERIALS AND METHODS

On 20 November 2020, we learned that our −80°C ultracold freezer, which stored many of our soil samples, malfunctioned, resulting in internal temperatures of 25°C for up to 1 week, thereby thawing all freezer contents. We took this opportunity to investigate how this short thawing event affected the compromised unextracted soil samples stored at −80°C, compared to the multi-year storage of extracted DNA stored at −20°C, which remained unaffected by the freezer failure. This allowed us to test whether freezer failure affected bacterial vs fungal resilience, as well as our ability to determine treatment effects and ultimately assess the viability of using these samples for long-term temporal studies. We selected 20 previously analyzed soil samples that had been collected from burned vs unburned plots at 25, 34, 67, 95, and 286 days after the 2018 Southern California Holy Fire, selecting 2 burned and 2 unburned plots for each of the 5 timepoints (23). The sequence files from these 20 soil samples, which never experienced a thaw event (“original” samples), were used to compare with new sequencing data generated from the soil samples that had been stored in the compromised −80°C freezer that thawed (“thawed” samples). We also resequenced extracted DNA that had been stored in an uncompromised −20°C freezer, with no buffer, for approximately 2 years (“frozen” samples). Samples from the thawed and frozen classification were selected to match the same timepoints as the original samples. Additional information on storage types is provided in Table S1. Site-specific details, including soil moisture, pH, and soil types, are available in Table S2.

### DNA extraction, sequencing, and bioinformatic and statistical analysis

In the summer of 2019, we extracted DNA from original soil samples stored at −80°C for 1 month to 1 year. These samples did not undergo any thawing events prior to being analyzed and have been published in our original study (23) and served as control samples. We selected a subset of 20 samples from these previously sequenced samples as frozen DNA samples for reanalysis. In October 2021, we extracted DNA from the 20 soil samples that had experienced the short thaw from the freezer malfunction, using the same methods as the original and frozen samples with Qiagen DNeasy PowerSoil Kits following the manufacturer’s protocol, as was done in the original study (23). We then used the same methods as had been used for the original samples to PCR amplify and sequence with Illumina MiSeq the 16S and ITS2 regions of the thawed and frozen DNA samples (23). We performed bioinformatics in QIIME2 (24) on all 60 samples (“original” vs “frozen” vs “thawed”) and performed downstream analyses on bacterial ASV tables rarefied to 6,493 sequences/sample and fungal (5,786 sequences/sample) as previously published (23).

Statistical analyses were performed in R (version 4.1.2), and all scripts can be accessed on GitHub (https://github.com/pulidofabs/Freeze-Thaw-FreezerMalfunction). We tested for correlations among bacterial vs fungal alpha diversity from the original vs frozen vs thawed samples using cor.test function in R with Pearson correlations (25). We further tested for correlations among bacterial vs fungal beta diversity among the treatments with mantel correlations in the Vegan package (26) using square-root transformed, Bray-Curtis dissimilarity matrices. We then assessed how the storage types affected ecological inference based on our main treatment effects of burned vs unburned plots. For species richness, we tested treatment effects using the lme4 package (27) controlling for nestedness level of plot and time since fire. For microbial composition, treatment effects were tested using PERmutational Multivariate ANalysis of Variance (PERMANOVA) as employed by the adonis2 function in vegan (21). We tested how the major taxonomic groups assessed in burned vs unburned plots varied in original vs frozen vs thawed with relative abundance plots created in ggplot2 (28) as had been done on the original study (23). Moreover, to test whether there were taxa that were differentially abundant between the storage types, we performed DESeq analysis between original vs thawed, original vs frozen, and thawed vs frozen samples.

## RESULTS

### Resilience of bacterial and fungal alpha diversity

**Fig 1:**
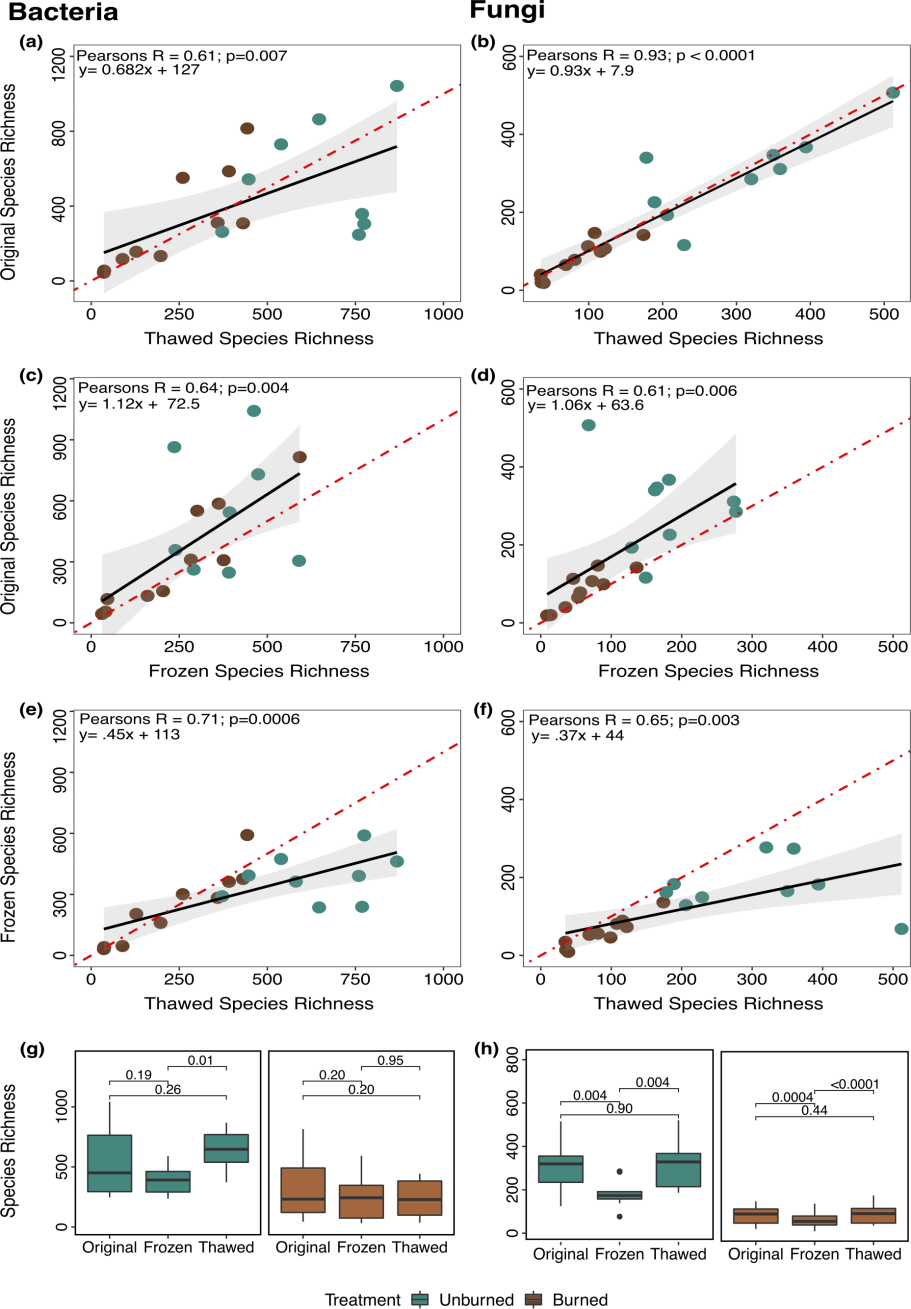
Comparison of bacterial and fungal species richness in unburned (blue-green) vs burned (brown) plots under different conditions: original, frozen, and thawed samples for bacteria (left panel) and fungi (right panel). Pearson correlations depict relationships between richness measures for (**a and b**) "original vs thawed” samples, (**c and d**) "original vs frozen,” and (**e and f**) “frozen vs thawed" samples. (**g**) bacterial and (**h**) fungal species richness comparison between conditions, within treatments. Black line represents the regression line, and red dashed line represents the 1:1 line. Boxplots represent the range where the middle 50% of all values lie, with the lower end of the box representing the 1st quartile (25th percentile) and the upper end representing the 3rd quartile (75th percentile).

In all cases, richness was strongly and significantly positively correlated to the original samples (Pearson’s *R* > 0.6) for both bacteria and fungi (Fig. 1). Moreover, bacterial (Fig. 1a) and fungal (Fig. 1b) richness were highly resilient to soil thawing, with both taxonomic groups largely maintaining the original species richness. Overall, storing soils at −80°C regardless of freezer malfunction is more robust for fungal richness (Pearson’s *R* = 0.93; Fig. 1b) than storing frozen DNA at −20°C (Pearson’s *R* = 0.61; Fig. 1d), whereas both conditions had equivalent impacts on bacterial richness (Pearson’s *R* = 61–64; Fig. 1a and c). The correlation between frozen and thawed conditions was slightly higher for bacterial (Pearson’s *R* = 0.71; Fig. 1e) than fungal species richness (Pearson’s *R* = 0.65; Fig. 1f).

Even though soil stored at −80°C experienced a short thaw, soils were more resilient than storage of extracted DNA in an uncompromised −20°C freezer. Indeed, there were never significant differences among median per sample richness for original vs thawed samples for either bacteria (Fig. 1g) or fungi (Fig. 1h) for either treatment condition. In contrast, DNA that had been extracted and stored in an uncompromised −20°C freezer for up to 2 years yielded significantly lower fungal richness than original or thawed in both burned and unburned soils (Fig. 1h) and lower bacterial richness than thawed samples in unburned soils (Fig. 1g; Table S3).

### Resilience of bacterial and fungal beta-diversity

**Fig 2:**
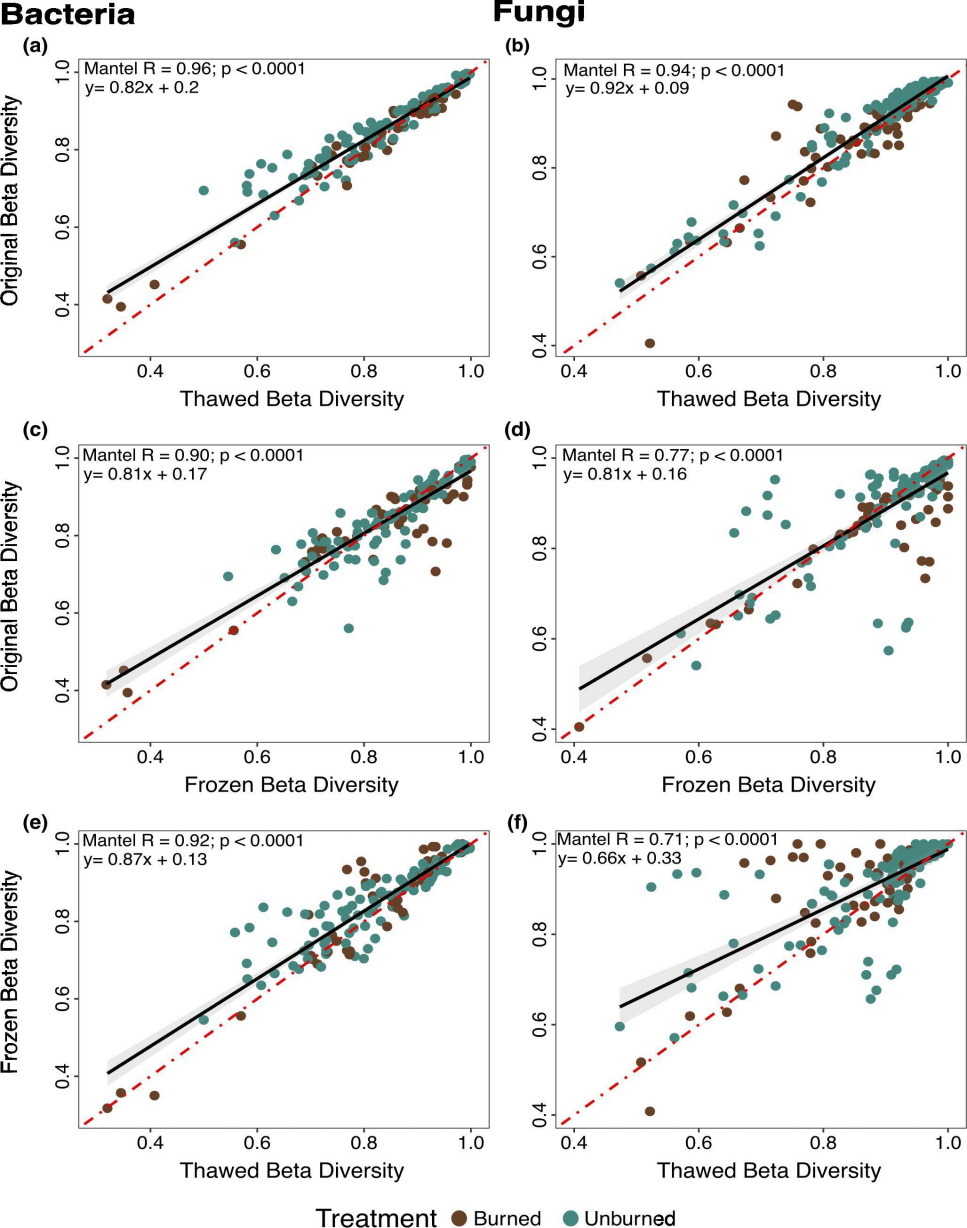
Comparison of bacterial (left panel) and fungal (right panel) beta-diversity as assessed by Bray-Curtis dissimilarity matrices. Mantel correlations compare the beta-diversity of (a) bacterial and (b) fungal original vs thawed, (c) bacterial and (d) fungal original vs frozen, and (e) bacterial and (d) fungal frozen vs thawed. Color denotes original treatment, with burned areas represented in brown and unburned areas in blue-green. Black line represents the regression and dashed red line the 1:1 line.

Beta-diversity is robust to thawing and freezing for both microbial groups, with strong positive correlations between original and thawed soil samples for both bacteria (*R* = 0.96; Fig. 2a) and fungi (*R* = 0.94; Fig. 2b). Bacterial beta-diversity also showed strong positive correlations among original and frozen samples (*R* = 0.90; Fig. 2c) and frozen vs thawed samples (*R* = 0.92; Fig. 2e). Fungal beta-diversity was also strongly positively correlated but was more sensitive than bacteria with less strong correlations among original and frozen (*R* = 0.77; Fig. 2d) and frozen and thawed conditions (*R* = 0.71; Fig. 2f). Notably, fungi from unburned plots subjected to long-term DNA storage at −20°C appeared to be more sensitive to changes than fungi from burned plots (Fig. 2d). Plotting the NMDS of individual samples collected at each timepoint revealed nearly indistinguishable effects of either freezing or thawing for bacteria (Fig. S1) and fungi (Fig. S2) at nearly every time point with frozen samples demonstrating the most differences (Fig. S2). These results suggest that while Bray-Curtis dissimilarity for both bacteria and fungi demonstrated high resilience to storage effects, bacterial composition exhibited greater resilience compared to fungi across all storage types (Fig. 2).

### Impacts of storage on ecological inferences between treatment and across time

**Fig 3:**
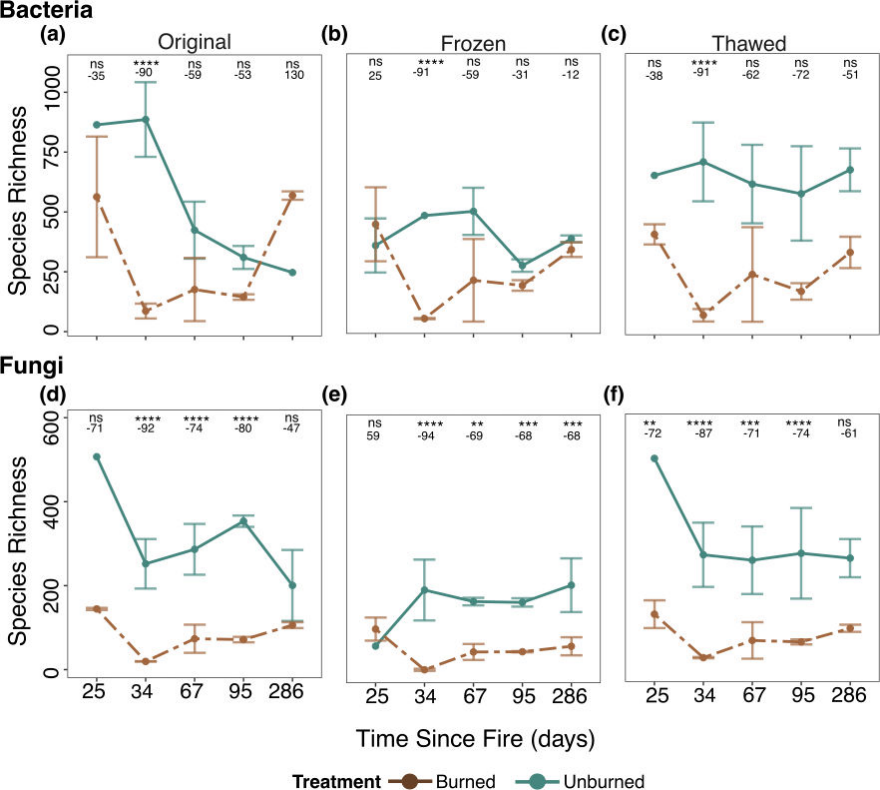
Effects of treatment (unburned in blue-green vs burned in brown) on mean bacterial and fungal species richness observed per time point for bacteria (top panels) and fungi (bottom panels) across original, frozen, and thawed storage types. Points represent per sample mean and lines represent standard error. Significance is indicated with asterisks (ns, not significant; *, *P* < 0.05; **, *P* < 0.01; ***, *P* < 0.001; ****, *P* < 0.00001), and numbers indicate the percent change between the burned and unburned samples at each time point.

Comparison between burned and unburned plots across storage types indicated that ecological inferences and the detection of treatment effects (richness averaged across time points) for fungi remained unaffected by freezing or thawing (Fig. S3). However, bacterial treatment effects were only detected in the thawed samples (Fig. S3c). For fungal richness, burned samples consistently exhibited a reduction of 66%–72% compared to unburned samples regardless of storage type (Fig. S3d and e). In contrast, thawing amplified the difference in bacterial richness between burned and unburned plots, increasing the reduction from 43% (*P* = 0.09; Fig. S3a) in the original samples to a significant reduction of 63% in the thawed samples (*P* = 0.001; Fig. S3c). Treatment differences over time were also largely unaffected by storage type (Fig. 3). For bacteria, there were only significant richness reductions in burned compared to unburned samples at 44 days post-fire in all storage conditions (original, frozen, thawed); however, the shapes of the curves vary slightly by storage type (Fig. 3a through c). For fungi, richness was significantly lower in burned compared to unburned samples at 3 of the 5 timepoints in original (Fig. 3d) vs 4 of the 5 timepoints for frozen (Fig. 3e) and thawed conditions (Fig. 3f). Moreover, the shapes of the curves for fungal richness are largely indistinguishable between original and thawed samples (Fig. 3d and f). However, there are slight differences observed at the first time point in frozen samples (Fig. 3e).

**Fig 4:**
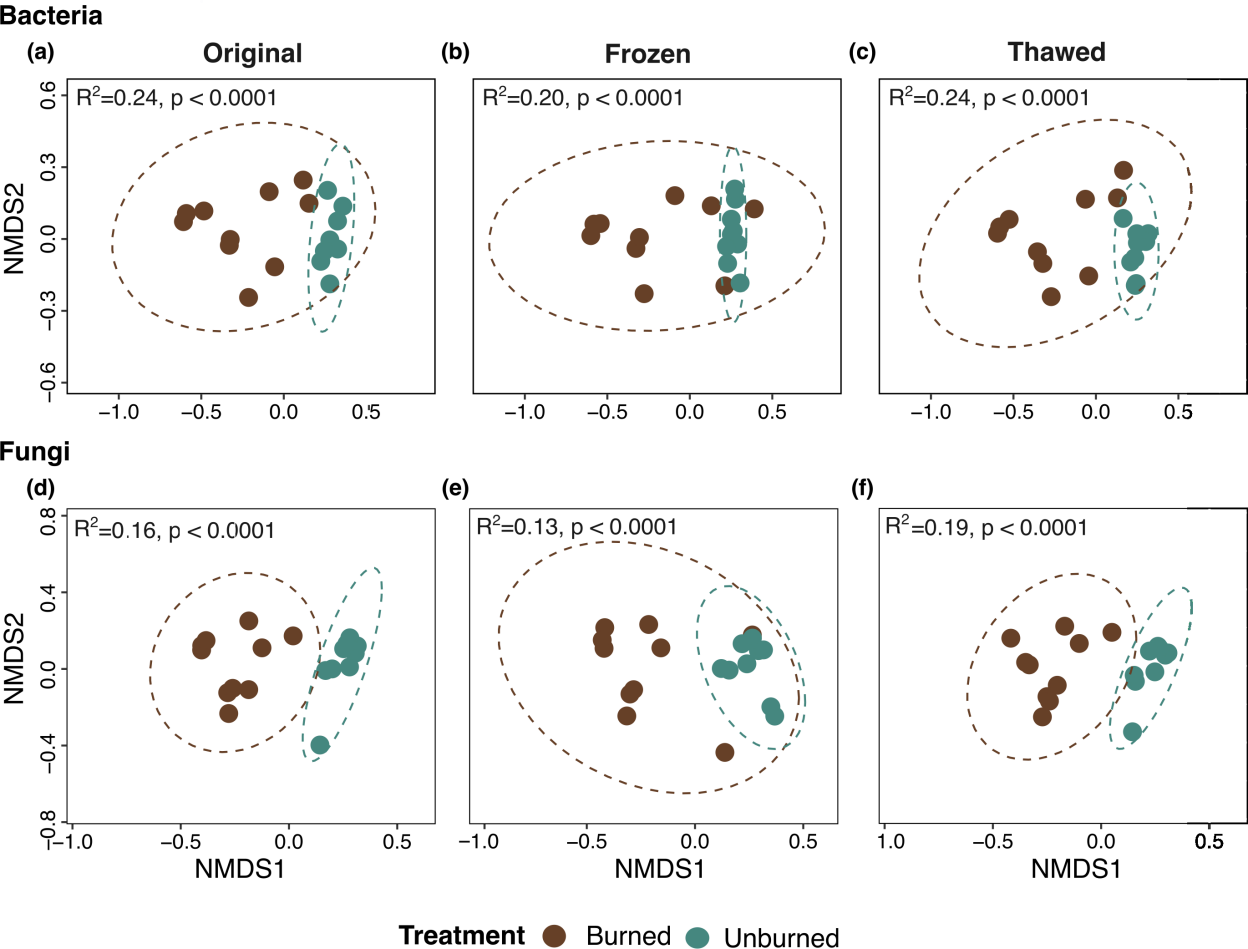
NMDS ordinations demonstrating effects of treatment (unburned in blue-green vs burned in brown) on microbial community composition across storage types. Impacts of fire on bacterial community composition in (a) original, (b) frozen, and (c) thawed samples. Impacts of fire on fungal community composition in (d) original, (e) frozen, and (f) thawed storage types. Ellipses represent the 95% confidence interval for each treatment based on Bray-Curtis dissimilarity. Significance based on Adonis.

Differences in community composition among treatments were unaffected by storage type (Fig. 4). For bacteria, beta-diversity was always significantly different in burned vs unburned samples (*P* < 0.0001) with identical *R*^2^ values for original and thawed (*R*^2^ = 0.24) and slightly lower *R*^2^ in frozen (*R*^2^ = 0.20; Fig. 4a through c). Similarly for fungi, beta-diversity was always significantly different in burned vs unburned samples across all storage types (*P* < 0.0001) with *R*^2^ ranging from 0.13 to 0.19 (Fig. 4d through f).

### Burned communities are more resilient to change in dominance than unburned communities

**Fig 5:**
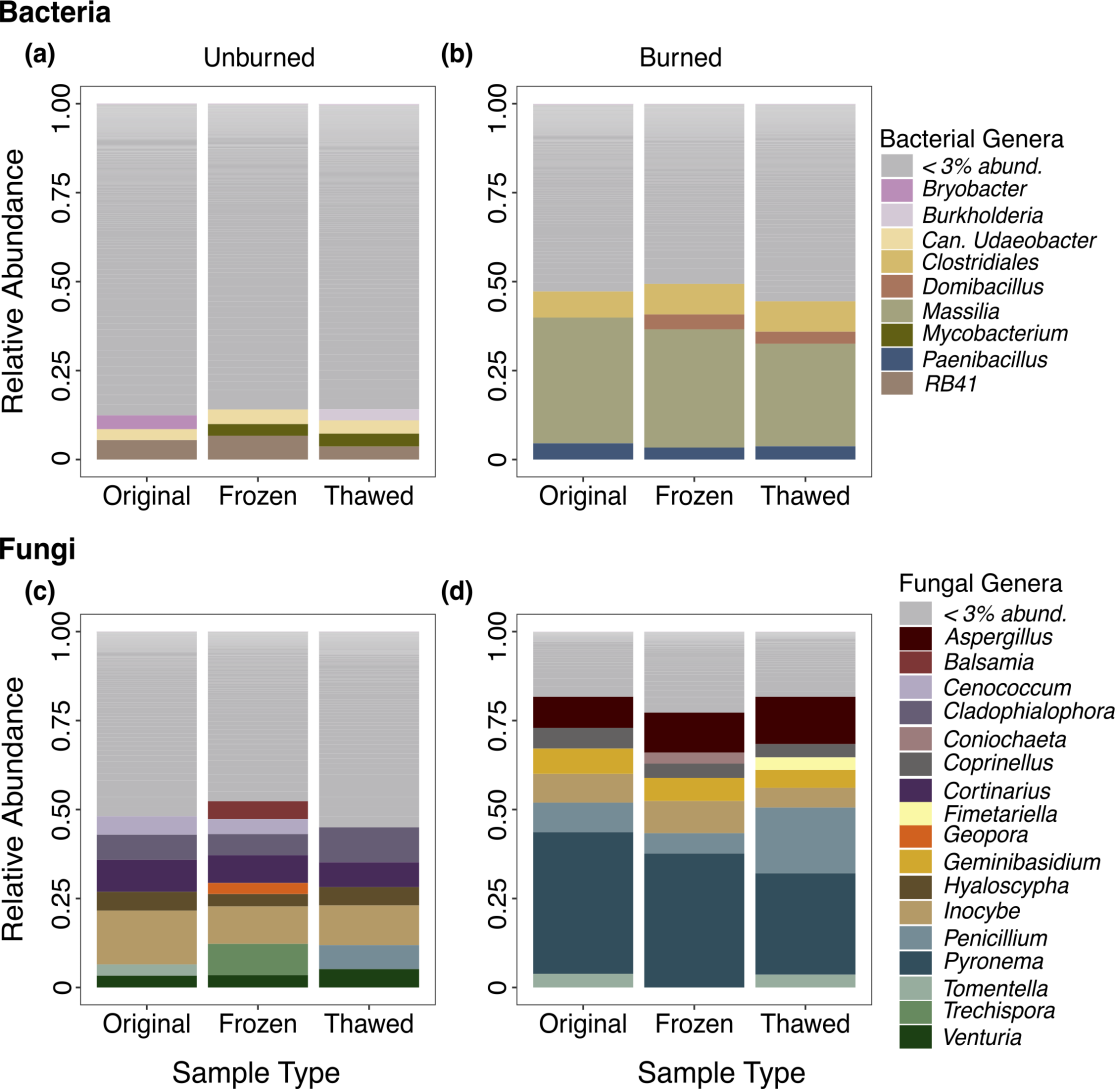
Taxonomy plots of dominant bacteria in (a) unburned and (b) burned plots and fungi in (c) unburned and (d) burned plots for original vs frozen vs thawed storage types. Genera that are greater than 3% relative sequence abundance are displayed in various colors, whereas genera <3% relative sequence abundance are summarized in gray for visualization.

Storage differences did not substantially alter the taxonomic composition of abundant genera for bacteria or fungi (Fig. 5). Similar to our findings from our original study (23), fire drastically altered the composition and dominance of bacteria and fungi with burned plots exhibiting higher dominance (Fig. 5b and d) than unburned plots (Fig. 5a and b). The taxonomic composition of genera with relative abundance above 3% (Fig. 5) and 1% (Fig. S4) from each storage type (original, frozen, or thawed) were similar for both burned and unburned communities.

Unburned bacterial samples shared 50% (323) of ASVs across storage types, while burned samples shared 48% (280) (Fig. S5a and b). Fungal samples showed similar trends, sharing 56% (179 ASVs) and 39% (78 ASVs), respectively (Fig. S5b and c). Dominant taxa remained detectable and stable after thawing and long-term DNA storage (Fig. 5). Consistent with the original study (23), bacterial RB41 dominated the unburned plots (Fig. 5a), while *Massilia* was prevalent in the burned plots (Fig. 5b). Thawed samples preserved a robust representation of the original community, with bacteria sharing 62% of ASVs with unburned and 58% with burned samples, while fungi shared 66% and 50%, respectively (Fig. S5a and b). In contrast, bacterial frozen samples shared 54%–55% of ASVs with original samples, while fungal frozen samples shared only 44% (unburned) and 62% (burned). Although unique ASVs were present in all storage types, they were more abundant in burned samples, comprising an average of 11% of the bacterial community and 14% of the fungal community across all storage types (Fig. S5c and d). However, despite the presence of unique taxa in every storage type, DESeq analysis revealed no significantly differentially abundant taxa in any comparison (original vs thawed, original vs frozen, and thawed vs frozen) at *P* < 0.05.

## DISCUSSION

Microbiome research relies heavily on freezer reliability for sample preservation, yet technological failures can jeopardize valuable data sets. In this study, we demonstrate that not all is lost when an unforeseen freezer failure occurs. Notably, correlations remained strongly and significantly positive for bacteria and fungi for both alpha (*r* > 0.6) and beta-diversity (*r* > 0.71) across all storage conditions. Bacterial richness was slightly more sensitive to the thaw event (*r* = 0.61) than fungal richness (*r* = 0.93) or bacterial (*R* = 0.94) or fungal (*R* = 0.96) composition, possibly due to the shorter bacterial DNA fragments, which are more stable during freeze-thaw cycles than longer fungal DNA fragments (26). Variations in DNA length and stability among bacterial species could also explain the sensitivity in bacterial richness. Nonetheless, ecological inferences based on treatment effects remained largely unchanged. We conclude that long-term storage of soil at −80°C is more robust than storage of extracted DNA at −20°C, even in the face of temporary ultracold freezer failure, since storing DNA at −20°C for 2 years led to decreased richness in both bacteria and fungi.

### Robustness of ecological inferences despite soil sample thawing

Overall, the fact that bacterial and fungal alpha metrics between the original and thawed samples were highly correlated suggest that ecological patterns and conclusions can be adequately reached. Indeed, we found consistent treatment effects on fungal species richness and community composition between the original and thawed samples, including similar decreases in richness between burned and unburned samples and alterations in community composition. Bacterial richness was more sensitive to thawing than fungal richness, which is corroborated by prior research on soils in a Chinese temperate forest (29). In our case, thawing caused by freezer failure amplified treatment effects when averaging across timepoints (Fig. S3) although treatment effects were not detected at most timepoints regardless of storage type when analyzed individually, likely because we only analyzed a subset of our original samples for the purposes of this analysis (Fig. 3). We speculate that thawing the soil magnified treatment effects because our soils came from chaparral shrublands that are adapted to arid environments (30, 31) and likely have evolved mechanisms for rapid response to increased soil moisture, such as those that occur during the thawing of soil, and thus explaining the increase in richness in unburned plots observed here. Indeed, prior research indicates that bacterial communities rapidly react to rain events (32).

### Long-term freezing negatively impacts microbial richness

Despite alterations in total species richness, ecological inferences based on our treatment effect remained the same across storage conditions for both bacteria and fungi (Fig. 3). However, our research indicates that storage types had similar impacts on bacterial richness (Pearson’s *R* = 61–64), while fungal correlations were lower in frozen samples (*r* = 0.61). In contrast, soil stored at −80°C showed a strong correlation with original samples (*r* = 0.93). This suggests that storing soil at −80°C is more robust for long-term data preservation, specifically for fungi, than freezing extracted DNA at −20°C, even if the −80°C freezer thaws for up to a week. This finding is particularly noteworthy, as the thawed samples were extracted from a different 0.25 g of soil than the original samples, while the frozen samples were derived from the exact same 0.25 g of soil as the original samples, and that DNA was then stored at −20°C. Freezing DNA at −20°C is a standard practice in microbiome work (3). Although prior research suggests that freezing DNA for up to 14 days has minimal impact on the community (15), extended freezing periods are often necessary for microbial studies. Our study demonstrated that long-term freezing of extracted DNA, spanning over 2 years, reduced bacterial and fungal richness in both burned and unburned samples, consistent with previous short-term freezing studies (16, 20, 21). Similarly, freezing at −20°C compromised the DNA integrity of bacteriophage lamda, with degradation increasing over the course of 1 year (33), suggesting that freezing overall can negatively impact microbiome DNA integrety. In this study, our extracted DNA was stored as per the Qiagen DNeasy PowerSoil Kits, which does not involve the use of any buffer (EDTA). However, studies suggest that physical shearing due to buffer type, influenced by pH and salt concentration, as well as freeze-thaw cycles (accessing DNA over time), can result in DNA degradation (34), such that 10% of the DNA is degraded with 1 thaw event and up to 75% after 20 thawing events (34, 35), suggesting that the lack of buffer in our samples is not the sole cause for the lack of DNA stability. This stability supports the use of long-term temporal analyses, where storing soil samples at −80°C and extracting DNA as needed may be more reliable than storing extracted DNA at −20°C for extended periods.

### Neither a short-term thaw nor long-Tterm freezing affect microbial community composition

The analysis of beta-diversity, crucial for understanding microbial community composition and treatment effects, remained highly robust across all sample storage methods. Our findings indicate that both long-term storage of extracted DNA in an uncompromised −20°C and re-extraction of soil stored at −80°C, even in the case of a failure and complete thaw, produced highly correlated beta-diversity metrics and yielded similar treatment effects to our original samples (23). This is consistent with a global meta-analysis study of soil freeze-thaw cycles of snow cover in various ecosystems (36).

Our study reveals the robustness of bacterial and fungal beta-diversity patterns across all storage types, with bacteria exhibiting greater resilience than fungi. Notably, the correlations between frozen and thawed fungal communities, and between original and frozen fungal communities, were lower compared to those observed for bacterial communities. This disparity may be attributed to the larger average base pair size of fungal DNA as compared to bacterial DNA, especially since smaller DNA sizes are known to confer improved stability against long-term freezing and increased freeze-thaw cycles (34). Despite lower correlations in fungal beta-diversity metrics to storage type, the ecological inferences of treatment effects based on community compositional differences across treatments remained the same. Together, our findings demonstrate the robustness of treatment effects, indicating that long-term microbial composition studies remain reliable even with short-term thawing or extended DNA storage at −20°C.

### Burned samples were more robust than unburned samples to freezing and thawing

We found particularly high robustness to storage conditions for our burned samples, building upon our original study that highlighted the lower diversity and heightened stability of burned microbial communities (23). Moreover, our findings support the notion that less diverse communities are inherently more stable due to reduced stochasticity (37). Importantly, our results suggest that soil samples with initially low biological diversity retain significant ecological information even after unforeseen thawing events, underscoring their enduring value and relevance. Additionally, the presence of stress-tolerant taxa within burned microbial communities (23, 38) could further confer resilience against rapid stressors such as unexpected thaw events. This resilience highlights the adaptability of microbial communities and emphasizes the importance of considering their potential responses to environmental perturbations in ecological research. Although unique taxa varied across storage types, on average they composed less than 9% of the unburned bacterial and fungal communities and 11%–14% of the burned community, respectively (Fig. S5). However, the distribution of dominant genera remained consistent across storage types, comprising over 50% of shared bacterial and 48% of fungal taxa. Thus, given that dominance is a key descriptor of burned and disturbed environments, and that DESeq analysis showed no significant differences between storage types, we can confidently conclude that biological inferences can, indeed, be made using thawed and frozen samples. Furthermore, these results align with studies indicating that dominant taxa are more resistant and resilient to environmental changes (39, 40). However, it is also possible that our particular soil samples might be more likely to be highly resistant to freezer failures. For example, the most dominant bacterial taxa in our burned samples, *Massilia*, is highly tolerant of abiotic stressors (41). The dominant fungal genera in our burned plots such as *Pyronema* produce resistant sclerotia (42) and fungal genera like *Penicillium* and *Aspergillus* are capable of producing resistant conidia (43), which may make them more likely to survive freezer failure.

### Conclusions

Here, we compared the ecological implications of an unexpected −80°C freezer failure (~1 week) and long-term DNA storage at −20°C (2 years) on soil microbiome studies and our ability to properly assess community dynamics and treatment effects for long-term monitoring studies. Our findings revealed high resilience of fungal richness and both bacterial and fungal beta-diversity to thawing of soil samples and long-term frozen DNA storage. The robust resilience of fungal richness facilitated biological inferences regarding treatment effects, regardless of storage type. However, for bacteria, although the patterns resembled those of the original samples, they appeared more pronounced in both frozen and thawed samples. In contrast, treatment effects were consistently detected for bacterial and fungal beta-diversity, suggesting that community composition is more resilient to small changes in environmental conditions. In conclusion, we suggest that, even in case of a short-term freezer failure, stored soil samples retain their usability for subsequent DNA based analysis, signifying promising potential for salvaging data in such situations.

## SUPPLEMENTAL MATERIAL

Supplemental material msphere.00603-24-s0001.docx Supplemental figures and tables.

ASM does not own the copyrights to Supplemental Material that may be linked to, or accessed through, an article. The authors have granted ASM a non-exclusive, world-wide license to publish the Supplemental Material files. Please contact the corresponding author directly for reuse.
