# Supplementary material for: Not all is lost: resilience of microbiome samples to freezer failures and long-term storage
Source: mSphere. 2024 Dec 20;10(1):e00603-24. doi: 10.1128/msphere.00603-24 (PMC11774026; doi:10.1128/msphere.00603-24)
Supplement: Supplemental material — Supplemental figures and tables. [file msphere.00603-24-s0001.docx]

**Figure S1:** Bacterial beta diversity between sample types (Frozen, Original and Thawed), based on Bray-Curtis dissimilarity for each individual sampling plot and timepoint (as denoted by the graph titles), where CNF refers to Cleveland National Forest (location of sampling), number refers to plot number, letter refers to cardinal direction (north east south west) and TX refers to timepoint. Significance based on Adonis.

**Figure S2:** Fungal beta diversity between sample types (Frozen, Original and Thawed), based on Bray-Curtis dissimilarity for each individual sampling plot and timepoint (as denoted by the graph titles),where CNF refers to Cleveland National Forest (location of sampling), number refers to plot number, letter refers to cardinal direction(north east south west) and TX refers to timepoint. Significance based on Adonis.

**Figure S3:** Original total treatment effects (burned vs unburned) averaged across all timepoints on bacterial and fungal species richness observed per sample type (original, thawed and frozen). Thawed samples are samples thawed between 1d to 1wk, frozen constitute frozen extracted DNA stored at -20C for 2 years and original are samples previously analyzed (controls). Richness based on rarefied observed ASV, and significance was tested using linear mixed models controlling for plot and time since fire. Negative percent value represents the percent decrease in richness between the burned and unburned samples.

**Figure S4:** Taxonomy plots of dominant bacteria in a) unburned and b) burned plots and fungi in c) unburned and d) burned plots for original versus frozen versus thawed storage types. Genera that are greater than 1% relative sequence abundance are displayed in various colors whereas genera <1% relative sequence abundance are summarized in grey for visualization.

**Figure S5:** Shared ASVs between unburned (left panels) and burned (right panel) sample types (original, thawed, and frozen) for bacteria (top) and fungi (bottom). Numbers represent total ASVs and percent of ASV as weighted by their relative abundance.

**Table S1**: Holy Fire Site-specific characteristics for the nine sampling plots (6 burned, 3 unburned) located within the 2018 Holy Fire that burned in the Cleveland National Forest in Riverside County. Soil pH and Soil moisture represent the average measurement for the first turnover event 25 days (T2) and the last turnover event 286 days (T8).

|  |  | **Soil pH** | | **% Soil Moisture** | | **Soil Taxonomic Class** |
| --- | --- | --- | --- | --- | --- | --- |
| **Site ID** | **Treatment** | **25 days (T2)** | **286 days (T8)** | **25 days (T2)** | **286 days (T8)** |  |
| CNF01 | Burned | 7.25 | 6.43 | 0.52 | 0.93 | Cieneba series; Loamy, mixed, superactive, nonacid, thermic, shallow Typic Xerorthents (Entisols) |
| CNF02 | Burned | 6.71 | 6.46 | 2.06 | 0.92 |  |
| CNF03 | Burned | 6.79 | 6.36 | 3.48 | 3.40 |  |
| CNF04 | Burned | 7.11 | 6.13 | 2.41 | 4.48 | Friant series; Loamy, mixed, superactive, thermic Lithic Haploxerolls (Mollisols) |
| CNF05 | Burned | 6.94 | 6.40 | 3.48 | 9.67 |  |
| CNF06 | Burned | 6.92 | 6.90 | 3.10 | 5.56 |  |
| CNF07 | Unburned | 6.1 | 6.32 | 7.65 | 11.21 |  |
| CNF08 | Unburned | 6.12 | 5.74 | 4.85 | 3.30 |  |
| CNF09 | Unburned | 6.18 | 5.98 | 4.46 | 4.58 |  |

**Table S2:** Sample type (original, frozen and thawed) storage and freeze-thaw information for years prior to thaw event and after thaw event.

| **Sample Type** | **Treatment** | **Storage condition** | **Experienced Freeze/Thaw** | **Storage Time Before Sequencing** | **Extraction Year** | | **Sequencing Year** |
| --- | --- | --- | --- | --- | --- | --- | --- |
| Original | Original (previously sequenced & published; Pulido et al., 2023) | Soil Frozen -80C prior to DNA extractions in 2019. | No | 1 month- 1 year | Summer 2019 | 2019 | |
| Frozen | Frozen -20C Extracted DNA from previously sequenced and published samples | Extracted DNA 2019 and stored in -20C until PCR and sequencing | No | 2 years | Oct-21 | 2021 | |
| Thawed | Thawed soils | Soil Frozen -80C | Yes | 2 years | Oct-21 | 2021 | |
|  | | | | | | | |

**Table S3**: Percent change in species richness between sample type (original, frozen and thawed) for bacterial and fungal communities for burned and unburned treatment (independently). All comparison are made against original sample type, a negative signifies a percent decrease.

| **Organism** | **Treatment** | **Sample Type** | **Mean Richness** | **Percent decrease** |
| --- | --- | --- | --- | --- |
| Bacteria | Burned | Original | 307.60 | 0.00 |
|  |  | Frozen | 239.60 | -22.11 |
|  |  | Thawed | 237.10 | -22.92 |
|  | Unburned | Original | 543.88 | 0.00 |
|  |  | Frozen | 382.11 | -29.74 |
|  |  | Thawed | 639.78 | 17.63 |
|  |  |  |  |  |
| Fungi | Burned | Original | 83.00 | 0.00 |
|  |  | Frozen | 59.20 | -28.67 |
|  |  | Thawed | 87.90 | 5.90 |
|  | Unburned | Original | 299.11 | 0.00 |
|  |  | Frozen | 176.56 | -40.97 |
|  |  | Thawed | 304.11 | 1.67 |
|  | | | | |
